# Comparison of 10% vs. 30% Velocity Loss during Squat Training with Low Loads on Strength and Sport-Specific Performance in Young Soccer Players

**DOI:** 10.3390/sports12020043

**Published:** 2024-01-30

**Authors:** Andrés Rojas-Jaramillo, Gustavo León-Sánchez, África Calvo-Lluch, Juan José González-Badillo, David Rodríguez-Rosell

**Affiliations:** 1 Research Group of Sciences Applied to Physical Activity and Sport, Universidad de Antioquia, Medellín 050010, Colombia; andres.rojasj@udea.edu.co; 2 Antioqueño Sports Research Center (CINDA) Indeportes, Medellín 050010, Colombia; gsanchez@indeportesantioquia.gov.co; 3 Department of Sport and Computer Science, Universidad Pablo de Olavide, 41013 Sevilla, Spain; acalllu@upo.es; 4 Physical Performance & Sports Research Center, Universidad Pablo de Olavide, 41013 Sevilla, Spain; jjgbadi@gmail.com; 5 Research, Development and Innovation (R&D+i) Area, Investigation in Medicine and Sport Department, Sevilla Football Club, 41005 Seville, Spain

**Keywords:** velocity-based resistance training, kicking ball speed, jump performance, training volume, high-speed actions, training specificity

## Abstract

The aim of this study was to compare the effects of two velocity-based resistance training (RT) programs using moderate loads (45–60% 1RM) but different magnitudes of velocity loss (VL) limits (10% vs. 30%) on the changes in physical performance in young soccer players. Twenty young soccer players were randomly allocated into two groups: VL10% (n = 10) and VL30% (n = 10). All participants were assessed before and after the 8-week RT program (twice a week) involving the following tests: 20 m running sprint (T20), countermovement jump (CMJ), kicking a ball (KB), and progressive loading test in the full squat (SQ) exercise. The RT program was conducted using only the SQ exercise and movement velocity was monitored in all repetitions. Significant ‘time × group’ interaction (*p* < 0.05) was observed for sprint performance, KB and 1RM in the SQ exercise in favor of VL10%. No significant changes between groups at post-test were observed. The VL10% resulted in significant (*p* < 0.05–0.001) intra-group changes in all variables analyzed, except for KB, whereas VL30% only showed significant (*p* < 0.05) performance increments in a sprint test and 1RM in the SQ exercise. The percentage of change and the intra-group’s effect size were of greater magnitude for VL10% in all variables analyzed compared to VL30%. In conclusion, our results suggest that, for non-trained young soccer players, squat training with low to moderate relative loads and 10%VL is sufficient to elicit significant increases in muscle strength and sport-specific actions compared to 30%VL in the set.

## 1. Introduction

Resistance training (RT) has been shown to have a beneficial effect on improving high-speed actions that are crucial for performance in soccer players, including vertical jump, acceleration capacity, change of direction ability, repeated sprint ability, or kicking ball (KB) speed [1,2,3,4,5]. However, the effectiveness of an RT program depends on how the different acute training variables (e.g., relative load; number of sets and repetitions; type and order of the exercises; recovery time between sets, repetitions, and exercises; frequency; and execution velocity) are manipulated [6].

Earlier studies involving soccer players have used high-load RT programs with training sets ending close to or at muscle failure [7,8]. Extensive literature has supported this approach as beneficial for maximizing strength gains and muscle hypertrophy [9,10]. However, RT with heavy loads and repetitions to failure presents some negative effects that limit its application in soccer players: (1) it tends to produce minimal gains or even detrimental effects on jump and sprint performance [7,8,10,11,12]; (2) it is associated with a high degree of fatigue [13,14] and leads to decreased performance of technical-tactical actions (passing and shooting accuracy) during the subsequent specific field training [15,16]; and (3) this type of training does not guarantee the greatest gains in physical performance [10,12]. In contrast to the aforementioned approach, there are numerous studies showing that RT (combined or not with plyometric training) with moderate or low loads, low number of repetitions per set, and performing each repetition at maximal intended velocity, produces significant improvements in lower-limb strength, vertical jump, running acceleration, the ability to repeat sprints, and KB speed in soccer players of different ages and categories [1,2,3,4,12]. Furthermore, this type of training has the advantage that it consumes less time, induces lower degrees of fatigue, and allows a much faster recovery [14].

Over the last decade, a focus has been placed on the use of movement velocity, from which a new approach (and paradigm) has emerged for monitoring RT, called velocity-based training [17]. According to this procedure, the training load for each session should be prescribed in terms of the velocity of the first (fastest) repetition (i.e., relative load) and the percentage velocity loss (%VL) in the set (i.e., volume) [18]. In this regard, several studies have analyzed the effect of velocity-based RT using different initial movement velocities and %VLs in the set in different exercises. In general, studies using the full-squat (SQ) exercise have shown that a low %VL (5–15%) induces less fatigue accumulation [18,19] and produces similar or even greater gains in strength, running speed, and jumping ability compared to a high magnitude of %VL in the set (>20%VL), regardless of the relative load used [5,10,11,12,19,20,21]. In addition, for the same %VL in the set, RT programs with lower relative loads tend to show greater gains in vertical jump and acceleration capacity [5,11,20,21]. Despite the importance that this new procedure has currently gained in the prescription and control of RT, to the best of our knowledge, there has been only one study using this approach in soccer players [5]. In this study [5], the effect of two RT programs (6 weeks, twice a week) with the same relative load (50–70% 1RM) but different %VLs in the set (15% vs. 30%) were compared in professional soccer players. The results revealed greater improvements in estimated 1RM and vertical jump for the group that trained with a VL of 15% compared to 30%, while no differences between groups were observed in sprinting or endurance capacity gains.

Despite these important findings, there is limited information on the manipulation of %VL in the set during RT using low to moderate relative loads and its possible effect on performance variables, including kicking ball speed, in young soccer players. Therefore, the aim of the present study was to compare the effects of two velocity-based RT programs in which low to moderate loads (45–60% 1RM) and different %VL limits (10% and 30%) were used to elicit changes in lower-limb strength, vertical jump, sprint ability, and kicking ball speed in young soccer players. Based on the previous studies using a velocity-based RT approach [5,10,11,12,20,21,22,23,24], we hypothesized that performing a squat training with 10% of VL in each training set will induce greatest gains in muscle strength, running acceleration, vertical jump, and KB speed performance compared to a squat training with 30% of VL in the set.

## 2. Materials and Methods

### 2.1. Participants

Twenty male young soccer players volunteered to participate in this study (Table 1). For a study of these characteristics, a total sample size of at least 20 participants (10 per group) was determined following a calculation for 85% statistical power, an alpha error of 0.05, and an effect size (ES) of 0.60 (G-Power version 3.1). The soccer players were members of the development program of a first-division professional soccer club in Colombia and competed in the first division of their age category. The players had no prior experience in systematic RT. All participants were injury-free for at least 3 months before participating in this study, and they had more than five years of playing soccer experience. None of the participants were taking drugs, medications, or dietary supplements known to influence physical performance during the time of the study. All participants participated in 100% of all training sessions. After an initial evaluation, players were matched according to their estimated 1RM in the SQ exercise (for more details, see Section 2.3.4) and then randomly assigned into two groups: 10% (VL10%, n = 10) or 30% (VL30%, n = 10). This study was conducted according to the Declaration of Helsinki and was approved by the Local Ethics Committee (ACEI 01-2021). After being informed of the purpose and experimental procedures, parental/guardian consents for all players involved in this investigation were obtained.

### 2.2. Study Design

A longitudinal and experimental study was used to analyze the effect of different velocity losses (VL10% vs. VL30%) during an 8-week velocity-based RT program on strength, jump, sprint, and kicking a ball performance in soccer players. For this, 20 young soccer players (VL10% = 10 and VL30% = 10) were assessed before (pre) and after (post) the training period using a sprint test in 20 m, a countermovement jump (CMJ) test, a kicking a ball test, and a progressive loading test in the full squat (SQ) exercise. The intervention was performed during the competitive season period (February–April). For the RT program, both experimental groups trained twice a week (Monday and Thursday) using only the SQ exercise, with moderate loads (45–60% 1RM). In addition, all the players continued with their conventional technical-tactical training. Both groups performed 4 sessions of soccer training per week and played a 90 min official match. The characteristics of the RT program for both experimental groups were identical. The only difference was the percentage of VL achieved in each training set: 10% (VL10%) or 30% (VL30%).

### 2.3. Testing Procedures

All evaluations were performed in a single session in the following order: 20 m sprint test, CMJ test, kicking a ball test, and progressive loading test in the SQ exercise. This sequence was arranged in an attempt to mitigate the potential impact of fatigue on the subsequent test performances. All participants carried out a general standardized warm-up consisting of 5 min of jogging on the court, 3 sets of progressively faster 30 m running accelerations, and followed by 5 progressively intensive jumps. Strong verbal encouragement was provided during all assessments to motivate participants to give maximal effort.

#### 2.3.1. Running Sprint Test

Players carried out 2 maximal 20 m running sprints (3 min rest) on a synthetic indoor running track and the best of both attempts was kept for analysis. The specific warm-up protocol consisted of one 40 m sprint at 80% effort, two 30 m sprints at 90% effort, and one 20 m sprint at maximal effort. Photocell timing gates (Newtest Oy, Oulu, Finland) were placed at 0, 10, and 20 m so that the times to cover 0–10 m (T10) and 0–20 m (T20) could be determined. A standing start, with the lead-off foot placed 1m behind the first timing gate, was used. The coefficients of variation (CV) for test–retest reliability for T10 and T20 were 1.6% and 1.4%, respectively. The intraclass correlation coefficients (ICCs) were 0.89 (95% confidence interval (CI): 0.70–0.96) for T10 and 0.93 (95% CI: 0.80–0.97) for T20.

#### 2.3.2. Countermovement Jump Test

A CMJ was performed with the subjects standing in an upright position on a contact platform (Chronojump Bosco System, Barcelona, Spain), with the hands on the hips to avoid arm swing. Then, participants performed a fast-downward followed by a fast-upward vertical movement as high as possible trying to reach the maximum possible vertical height. Five trials were completed with 45 s rest between each trial. The highest and lowest values were discarded, and the resulting mean of the 3 remaining jump values was kept for analysis. The specific warm-up consisted of 2 sets of 10 repetitions of the squat exercise without extra load (2 min rest), 5 CMJs at progressive intensity (20 s rest), and 3 maximal CMJs (30 s rest). The CV was 2.2% and the ICC was 0.99 (95% CI: 0.99–1.00).

#### 2.3.3. Kicking a Ball (KB) Test

Players performed a maximal velocity kick to a stationary ball placed in the penalty spot. The kick was performed with the dominant leg. A ball with a standard Colombia Federation of soccer size and inflation was kicked toward the goal. The players were asked to shoot the ball as hard and fast as possible. The initial distance of the player from the ball was 3 m. This distance was chosen to make an approach race before kicking the ball. Two shots were allowed for each player (1 min rest) and the maximum value was kept for the subsequent analysis. The peak speed of the ball was measured by a velocity speed radar gun (Busnell^®^, Overland Park, KS, USA) located 2 m from the stationary ball and pointed toward the ball according to the instruction manual. The CV was 2.8% and the ICC was 0.90 (95% CI: 0.73–0.97).

#### 2.3.4. Progressive Loading Test in the SQ Exercise

This test was performed on a Smith machine (SportsArt fitness, Mukilteo, WA, USA). The players performed the SQ from an upright position, descending (eccentric phase) in a continuous motion until the posterior thighs and calves made contact with each other. Then, they immediately reversed the motion and ascended back to the starting position. The players were required to always execute the concentric phase at a maximal intended velocity in all repetitions. The initial load was set at 15 kg for all players and was gradually increased in 10 kg increments until the mean propulsive velocity (MPV) was lower than ~0.60 m·s^−1^. During the test, 3 repetitions were executed for light (MPV > 1.10 m·s^−1^), 2 for medium (1.10 m·s^−1^ > MPV > 0.80 m·s^−1^), and only 1 for the heaviest (MPV < 0.80 m·s^−1^) loads. The interset rest was 3 min. The exact same progression of absolute loads was repeated in the pre- and post-test for each participant. Only the best repetition at each load, according to the criterion of fastest mean propulsive velocity (MPV), was considered for subsequent analysis. The following variables derived from this test were used for analysis: (i) the estimated 1RM calculated for each individual from the MPV attained against the heaviest load (kg) lifted in the progressive loading test, as follows (100 × load)/(−5.961 × MPV2) − (50.71 × MPV) + 117 [22]; (ii) average MPV attained against all absolute loads common to pre- and post-tests (AV); and (iii) MPV attained against 15 kg (MPV15), 25 kg (MPV25), 35 kg (MPV35), 45 kg (MPV45), 55 kg (MPV55), and 65 kg (MPV65). The velocity of each repetition was measured with a linear position transducer (Chronojump system, Barcelona, Spain).

### 2.4. Resistance Training Program

All players conducted a total of 16 velocity-based RT sessions over 8 weeks using only the SQ exercise. All training variables, including relative loads (45–60% 1RM), number of sets (three), recovery time between sets (three min) and sessions (~72–96 h), and training frequency (2 sessions per week) were the same for both experimental groups. The only difference between VL10% and VL30% was the magnitude of VL achieved in each training set. Table 2 shows in detail the descriptive characteristics of the RT program actually performed by soccer players. Relative loads were determined from the load–velocity relationship for the SQ. Thus, a target MPV to be attained in the first (usually the fastest) repetition of the first exercise set in each training session was used as an estimation of percentage of 1RM, as follows: ~1.24 m·s^−1^ (~45% 1RM), ~1.15 m·s^−1^ (~50% 1RM), ~1.08 m·s^−1^ (~55% 1RM), and ~1.00 m·s^−1^ (~60% 1RM) [22]. Once the load (kg) was adjusted, it was maintained for the three training sets. Volume in each training set was objectively determined through the magnitude of VL attained over the set [20]. Thus, the training set was terminated when the prescribed velocity loss limit was reached. Training sessions were performed using the free weight SQ exercise. All repetitions of all players during each session were recorded using a linear position transducer (Chronojump Bosco system). Players received immediate movement velocity feedback while being encouraged to perform each repetition at the maximal intended velocity. The RT sessions were performed 1.5 h before the soccer training session. During all training sessions, players carried out a general standardized warm-up which consisted of 5 min of jogging on the court, 3 sets of 20 m accelerations with progressive intensity, and finally 5 CMJ with progressive intensity.

### 2.5. Statistical Analysis

Standard statistical methods were used for the calculation of means and standard deviations (SDs). The normality of the distribution of variables at pre was examined with the Shapiro–Wilk test, and the homogeneity of variance across groups (VL10% vs. VL30%) was verified using Levene’s test. A one-way random effects model (model 2.1) ICC was used to determine relative reliability with a 95% confidence interval. Absolute reliability was reported using the CV. A 2 (group: VL10% vs. VL30%) × 2 (time: pre vs. post) factorial ANOVA with Bonferroni’s adjustment was used to analyze the differences between experimental groups. The intra-group effect sizes (ESs) were calculated using Hedge’s g, with the corresponding 95% confidence interval (95% CI). The ES thresholds for small, moderate, and large effects were 0.2, 0.5, and 0.8, respectively. Statistical significance was accepted at *p* < 0.05. The null hypothesis tests were performed using SPSS software version 26.0 (SPSS, Chicago, IL, USA).

**Table 2:** Descriptive characteristics of the squat training program performed by VL10% and VL30% groups.

Actually Performed	S1	S2	S3	S4	S5	S6	S7	S8	S9
VL (%)									
VL10%	10.6 ± 0.4	10.4 ± 0.5	10.7 ± 0.5	10.4 ± 0.4	10.2 ± 0.2	10.4 ± 0.3	10.3 ± 0.4	10.2 ± 0.3	10.4 ± 0.4
VL30%	30.3 ± 0.9	32.7 ± 6.7	30.5 ± 0.4	30.4 ± 0.8	31.1 ± 1.1	30.7 ± 0.4	30.7 ± 0.7	30.5 ± 0.9	31.2 ± 0.7
Average Nº Rep per set									
VL10%	5.2 ± 1.4	5.5 ± 1.6	7.4 ± 1.6	6.6 ± 1.8	5.5 ± 0.9	7.2 ± 2.0	6.8 ± 0.6	7.9 ± 2.6	5.6 ± 1.0
VL30%	13.9 ± 5.3	13.5 ± 4.0	17.5 ± 7.3	14.7 ± 6.5	15.3 ± 7.5	15.0 ± 4.5	16.3 ± 6.8	16.3 ± 7.9	14.6 ± 6.0
Reference rep’s MPV (m·s^−1^)									
VL10%	1.21 ± 0.01	1.21 ± 0.01	1.21 ± 0.01	1.21 ± 0.01	1.15 ± 0.01	1.15 ± 0.01	1.15 ± 0.01	1.15 ± 0.01	1.08± 0.01
	(~46.8% 1RM)	(~46.9% 1RM)	(~46.7% 1RM)	(~46.7% 1RM)	(~50.7% 1RM)	(~50.8% 1RM)	(~50.8% 1RM)	(~50.8% 1RM)	(~49.9% 1RM)
VL30%	1.21 ± 0.01	1.21 ± 0.01	1.21 ± 0.01	1.21 ± 0.01	1.15 ± 0.01	1.15 ± 0.02	1.15 ± 0.01	1.15 ± 0.01	1.08 ± 0.01
	(~46.9% 1RM)	(~46.7% 1RM)	(~46.7% 1RM)	(~46.7% 1RM)	(~50.6% 1RM)	(~50.8% 1RM)	(~50.8% 1RM)	(~50.9% 1RM)	(~55.6% 1RM)
Actually Performed	S10	S11	S12	S13	S14	S15	S16		Overall
VL (%)									
VL10%	10.2 ± 0.4	10.8 ± 0.6	10.5 ± 0.5	10.2 ± 1.0	10.8 ± 1.0	11.5 ± 1.1	10.8 ± 0.8		10.5 ± 0.7
VL30%	31.3 ± 0.6	31.1 ± 0.5	31.5 ± 0.8	30.5 ± 0.7	30.6 ± 0.7	30.6 ± 0.7	30.5 ± 1.0		30.9 ± 1.8
Average Nº Rep per set									
VL10%	6.4 ± 2.0	6.3 ± 2.2	6.0 ± 1.0	5.9 ± 1.6	6.2 ± 1.2	9.1 ± 2.6	6.8 ± 2.1		6.5 ± 2.0
VL30%	13.4 ± 5.5	12.3 ± 5.0	17.7 ± 4.9	14.4 ± 5.2	18.2 ± 4.2	16.2 ± 4.3	17.9 ± 5.2		16.5 ± 8.5
Reference rep’s MPV (m·s^−1^)									
VL10%	1.08 ± 0.01	1.08 ± 0.01	1.08 ± 0.01	1.01 ± 0.01	1.01 ± 0.01	1.01 ± 0.02	1.01 ± 0.01		1.11 ± 0.08
	(~55.6% 1RM)	(~55.4% 1RM)	(~55.6% 1RM)	(~59.5% 1RM)	(~59.6% 1RM)	(~59.4% 1RM)	(~59.5% 1RM)		(~53.1% 1RM)
VL30%	1.08 ± 0.02	1.08 ± 0.01	1.08 ± 0.01	1.01 ± 0.01	1.01 ± 0.01	1.01 ± 0.01	1.01 ± 0.01		1.11 ± 0.08
	(~55.5% 1RM)	(~55.5% 1RM)	(~55.7% 1RM)	(~59.7% 1RM)	(~59.6% 1RM)	(~59.5% 1RM)	(~59.9% 1RM)		(~53.1% 1RM)

Abbreviations: The groups (n = 10 each) trained with different percent velocity loss (VL) in each set = 10% (VL10%) and 30% (VL30%). VL = velocity loss; S = session; MPV = mean propulsive velocity; 1RM = one-repetition maximum; Nº Rep = number of repetitions per set.

## 3. Results

### 3.1. Training Program

**Figure 1:**
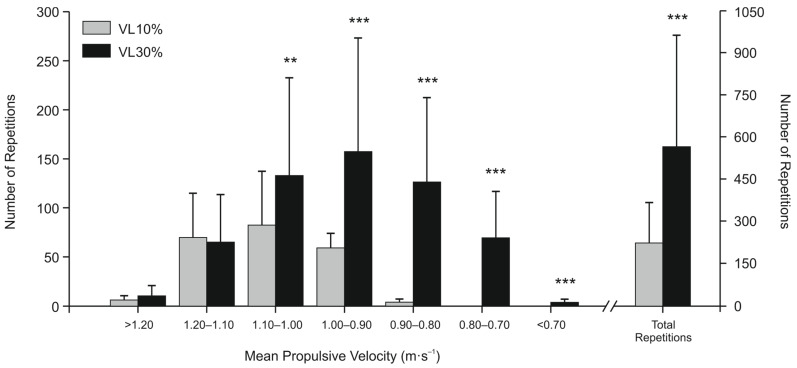
Number of repetitions performed in each velocity range and total number of repetitions completed by both experimental groups. Data are mean ± SD. Statistically significant differences between groups: ** *p* < 0.01, *** *p* < 0.001. VL10%: Group with 10% velocity loss (n = 10), VL30%: Group with 30% velocity loss (n = 10). Warm-up repetitions are excluded.

No significant differences between groups were observed for the fastest MPV of the first set (i.e., relative load, %1RM) in any session. Players in VL30% performed significantly (*p* < 0.001) more average total repetitions with the maximal scheduled load in each training session (565.4 ± 397.8) than those in the VL10% (221.5 ± 144.9). Soccer players in VL10% trained at a significantly (*p* < 0.001) faster mean velocity than those in VL30% (1.05 ± 0.08 m·s^−1^ vs. 0.95 ± 0.13 m·s^−1^, respectively). The VL30% performed a significantly greater number of repetitions than VL10% when MPV was <1.10 m·s^−1^, whereas no significant differences between groups were observed for MPV > 1.10 m·s^−1^ (Figure 1).

### 3.2. Physical Performance Variables

**Figure 2:**
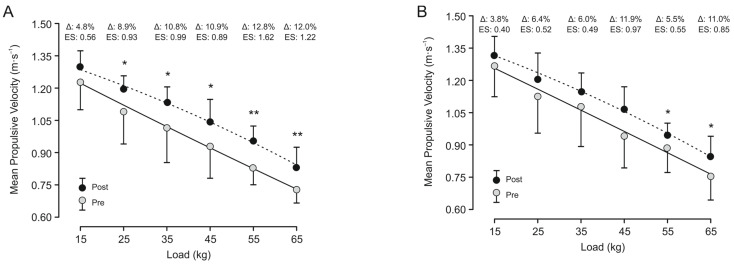
Load–velocity curves in the full-squat exercise for VL10% (**A**) and VL30% (**B**) before and after an 8-week training period. Data are mean ± SD. Statistically significant differences within group: * *p* < 0.05, ** *p* < 0.01. Note: only the loads that were executed by all the participants in both tests were analyzed.

Data for all variables analyzed were homogeneous and normally distributed. There were no significant differences between groups (VL10% vs. VL30%) at baseline in any of the variables studied. Significant ‘time × group’ interaction (*p* < 0.05) was observed for T10, T20, KB, and 1RM in favor of VL10% (Table 3). No significant changes between groups at post-test were observed in any variable (T10 *p* = 0.057; T20 *p* = 0.306; KB speed *p* = 0.065; CMJ *p* = 0.227; RM *p* = 0.964; AV *p*= 0.209). However, VL10% resulted in significant (*p* < 0.05–0.001) pre–post-changes in all variables analyzed, except for (*p* = 0.448) and MPV15 (*p* = 0.213), while VL30% only showed significant increments in T20, 1RM, MPV55, and MPV65. In addition, VL30% showed a significant (*p* < 0.05) performance loss in the variable KB speed. The percentage of change and the intra-group ESs were of greater magnitude for VL10% in all variables compared to VL30% (Table 3 and Figure 2).

## 4. Discussion

The main findings of the present study were that the VL10% resulted in significant gains in all variables assessed, except for KB, whereas the VL30% group only showed significant performance increases in the T20, MPV55, MPV65, and estimated 1RM. In addition, although no significant differences between groups were observed in any variable at post, our results showed a significant ‘time × group’ interaction (*p* < 0.05) in T10, T20, KB, and 1RM in favor of VL10%. In addition, the percentage of change and intra-group ES were greater for VL10% compared to VL30% in all variables. These results were especially noticeable considering that VL10% (221.5 ± 144.9 repetitions) performed, on average, approximately 40% of the total repetitions that VL30% performed (565.4 ± 397.8 repetitions). Therefore, in accordance with our initial hypothesis, our results suggest that, for non-trained young soccer players, RT programs with low to moderate relative loads and low %VL (10%) are sufficient to produce significant increases in muscle strength and sport-specific performance parameters compared to moderate to high %VL (30%) in the set.

Previous studies [5,10,11,20,23] and meta-analyses [12,24] comparing the effect of VBRT with different %VL in the set have shown similar results to those observed in the present study, regardless of the relative load used. Specifically, studies using only the SQ exercise during the training program have found that the magnitudes of VL in the set of 5% [21], 10% [11,20], or even 20% [10] produced similar or even greater increments in strength, jump, and sprint performance than higher %VL in the set (30% or 40–45%) [10,11,20]. Although these studies were conducted with RT-experienced subjects (usually sports science students) and not with soccer players or athletes, these results reinforce the idea that it is not necessary to apply heavy loads and repetitions to muscle failure to obtain gains in strength or physical performance, as has been postulated in numerous studies and position stands [9]. In addition, the objective in soccer players would be to apply an RT stimulus that produces long-term benefits on physical performance without reducing acute performance during subsequent field soccer training. Previous studies have indicated that RT involving heavy loads and repetitions per set ending near or to muscle failure (i.e., high %VL in the set) induced greater immediate and short-term deteriorations in jump, sprint, and soccer skill performance compared to lower repetitions or %VL in the set [15,16,18,19,25]. Thus, RT programs with light loads and low %VL appear to be more efficient because they allow players to obtain the same or greater improvements but with a lower degree of fatigue and better performance in subsequent field soccer training.

Two previous studies have compared RT programs only using the SQ exercise and similar %VL in the set (10% vs. 30%) based on changes in strength and physical performance [11,20]. Although the participants in these studies were not soccer players and the range of relative loads used was higher (55–70% 1RM and 70–85% 1RM), the results obtained were similar to those shown in the present study: VL10% groups obtained similar gains in strength (18–22% and 15–22% for VL10% and VL30%, respectively) but greater beneficial effects on jump (9–11% and 5–6% for VL10% and VL30%, respectively) and sprint performance (−1.5 to −2.4% and 0.4 to −1.8% for VL10% and VL30%, respectively) than VL30% [11,20]. As observed, the percentage of change in CMJ and estimated 1RM in the SQ exercise were practically identical to those obtained in our investigation (Table 3). However, the changes in T20 were much higher in the present study (−11.3% and −9.6% for VL10% and VL30%, respectively). These differences may be because (i) the participants in this study showed lower levels of initial performance in this variable; (ii) they were younger; and (iii) they had no prior experience in RT compared to previous studies. All these factors are related to a greater adaptation deficit and, therefore, have a greater predisposition to improve strength, velocity, and power output [2,26,27]. Despite these findings, to the best of our knowledge, only one study examined the effect of applying two VBRT programs differing only in the percentage of VL achieved in each training set (VL15% vs. VL30%) in soccer players [5]. In agreement with our results, Pareja-Blanco et al. [5] found that a VBRT program characterized by a low degree of fatigue (i.e., 15% VL and load ranging from 50–70% 1RM) was effective in inducing significant improvements in jump (∆: 8.9%; ES: 0.43) and 1RM strength (∆: 5.3%; ES: 0.45), although these gains were lower than those observed in our study. As indicated, these differences may probably be due to the higher level of initial strength and age of the participants and to the fact that they had previous experience in RT. In addition, given that in our study lower relative loads against similar %VL was used, the average training velocity in all training sessions was higher compared to Pareja-Blanco et al. [5]. This could allow a greater benefit (i.e., transfer) for this type of training on high-speed actions, such as the CMJ and sprint performance in 20 m [11,20,28].

With regard to the KB test, the VL10 group showed no significant change in the peak speed of the ball (∆: 1.0%; ES: 0.15), although the most relevant aspect was that the VL30% group showed a significant decrement (∆: −2.6%; ES: −0.40) in this variable after the RT program. To the best of our knowledge, there have been no studies analyzing the effect of RT programs using a velocity-based approach in young soccer players on change in KB speed. However, a recent study [4] with futsal players using an SQ training program with similar characteristics to those used in the VL10% group of our research showed significant increments (∆: 2.8%; ES: 0.52) in this variable. Similarly, other studies using RT programs with low-load, low-volume, and high-execution velocity in each repetition have also reported increments in kicking ball speed [29,30]. According to these previous studies, results of VL10% were not expected. In contrast, it appears that RT programs involving heavy-load resistance training (4–8 RM) [31] or high %VL in the set (e.g., 30% VL) may not produce positive adaptations or may even worsen ball speed performance. This could be because RT with these characteristics is related to a very low absolute muscle-shortening velocity during each training session, resulting in 1RM strength gains but not increments in the force applied during the short contact time between the foot and the ball during kicking (~10–12 ms) [32].

An escalating concern in contemporary soccer, prevalent among both adults and young players, is the soaring injury rates [33,34]. This situation appears to be partly linked to overtraining [35]. It seems that soccer players endure high training loads during specific field training, coupled with the demands of physical preparation (e.g., RT or analytical endurance training) and matches. These continuous and excessive demands could have adverse effects on physical performance and elevate the risk of injuries [35]. For this reason, one of the basic objectives for coaches and scientists should be to apply more efficient training programs, i.e., that allow obtaining the same or even greater technical–tactical and physical performance with a lower level of effort in each training session. Specifically, the results of the present study, along with those previously shown in other investigations [1,2,3,4,5,11,12], could be relevant for improving RT methodology for young soccer players. In this regard, contrary to what is recommended in some studies and position stands [9], coaches and strength and conditioning professionals for young soccer players should consider using lower %VL in the set (e.g., 10%) in RT programs aimed at improving high-speed actions in soccer young players because these degrees of fatigue provide greater beneficial effects on jump and sprint performance compared to VL in the set greater than 20%. In addition, low %VL in the set is associated with shorter training duration, lower degree of fatigue, minimized decline in technical-tactical performance in the subsequent specific field soccer training, and faster recovery times following RT sessions [13,14,18,19].

Despite the important findings observed, the present study has some limitations that need to be addressed. The main limitation could be related to the low number of participants in each group. In this regard, we cannot rule out a type II error when comparing the two types of training. In fact, some variables (e.g., T10, KB speed, and CMJ) showed between groups’ *p*-values close to 0.05, indicating that training with a lower %VL may result in more favorable adaptations to enhance performance in dynamic exercises than reported herein. Additionally, another potential limitation lies in the absence of load control during specific field soccer training. Lack of data (e.g., GPS) prevented the assessment of the players’ exertion levels during the intervention period. However, it is important to note that the possible “negative effect” that the specific field training load could have produced on the adaptations induced by RT was similar for both training groups.

## 5. Conclusions

RT programs using only the SQ with light to moderate loads (~1.21–1.00 m·s^−1^ or 45–60% 1RM) and low %VL (~10%) produced greater beneficial effects on CMJ, sprint performance, and lower-limb strength than the same RT program with a higher %VL (~30%) in the set in non-trained young soccer players. In addition, VL10% allowed maintenance of performance in KB speed, whereas players in VL30% showed a significant performance loss in this variable.

## Figures and Tables

**Table 1:** Characteristics of participants.

Groups	Weight (kg)	Height (m)	Age (years)
VL10%	73.3 ± 4.7	1.73 ± 0.05	16.3 ± 0.2
VL30%	73.6 ± 4.3	1.72 ±0.06	16.6 ± 0.3

Abbreviations: VL10% = Group training with 10% velocity loss in the set; VL30% = Group training with 30% velocity loss in the set.

**Table 3:** Changes in selected neuromuscular performance variables from pre- to post-training for each training group.

	VL10%				VL30%			
Variable	Pre	Post	% Δ	ES (95% CI)	Pre	Post	% Δ	ES (95% CI)
CMJ (cm)	38.2 ± 7.2	41.5 ± 5.8 *	8.0	0.51 (−0.02 to 1.03)	37.8 ± 6.2	38.83 ± 5.56	2.6	0.17 (−0.40 to 0.74)
T10 (s) ^†^	2.02 ± 0.08	1.79 ± 0.13 ***	−12.8	−2.06 (−3.34 to −0.78)	2.03 ± 0.06	1.84 ± 0.14 **	−10.8	−1.90 (−3.20 to −0.61)
T20 (s) ^†^	3.38 ± 0.14	3.04 ± 0.22 ***	−11.3	−1.85 (−2.93 to −0.77)	3.38 ± 0.13	3.09 ± 0.21 ***	−9.6	−1.72 (−2.83 to −0.61)
1RM (kg) ^†^	77.63 ± 14.72	99.75 ± 11.88 ***	22.2	1.65 (0.74 to 2.56)	81.1 ± 17.3	99.4 ± 12.1 ***	18.4	1.22 (0.45 to 1.99)
AV (m·s^−1^)	0.98 ± 0.08	1.11 ± 0.08 **	11.7	1.61 (0.08 to 3.14)	1.00 ± 0.08	1.11 ± 0.08 **	10.0	1.35 (0.26 to 2.44)
BS (km·h^−1^) ^††^	100.79 ± 7.25	101.79 ± 6.60	1.0	0.15 (0.02 to 0.27)	101.79 ± 4.92	99.18 ± 7.74 ***	−2.6	−0.40 (−0.94 to 0.13)

Data are mean ± SD. Abbreviations: The groups (n = 10 each) trained with different percent velocity loss (VL) in each set = 10% (VL10%) and 30% (VL30%). Pre = initial assessment; post = final assessment; ES = intra-group effect size; CI = confidence interval; 1RM = estimated one-repetition maximum in the full squat exercise; CMJ = countermovement jump height; T10 = 10 m sprint time; T20 = 20 m sprint time; AV = average MPV attained against all absolute loads common to pre- and post-tests in the squat progressive loading test; BS = kicking ball speed. Statistically significant “time × group” interaction = ^†^ *p* < 0.05, ^††^ *p* < 0.01. Intra-group significant differences from pre to post = * *p* < 0.05, ** *p* < 0.01, *** *p* < 0.001.

## Data Availability

The data presented in this study are available on request from the corresponding author. The data are not publicly available due to confidentiality.
